# Legacy of historic ozone exposure on plant community and food web structure

**DOI:** 10.1371/journal.pone.0182796

**Published:** 2017-08-10

**Authors:** M. Alejandra Martínez-Ghersa, Analía I. Menéndez, Pedro E. Gundel, Ana M. Folcia, Ana M. Romero, Jennifer B. Landesmann, Laura Ventura, Claudio M. Ghersa

**Affiliations:** 1 IFEVA, Facultad de Agronomía, Universidad de Buenos Aires, CONICET, Buenos Aires, Argentina; 2 Departamento de Producción Vegetal, Facultad de Agronomía, Universidad de Buenos Aires, Buenos Aires, Argentina; Pacific Northwest National Laboratory, UNITED STATES

## Abstract

Information on whole community responses is needed to predict direction and magnitude of changes in plant and animal abundance under global changes. This study quantifies the effect of past ozone exposure on a weed community structure and arthropod colonization. We used the soil seed bank resulting from a long-term ozone exposure to reestablish the plant community under a new low-pollution environment. Two separate experiments using the same original soil seed bank were conducted. Plant and arthropod richness and species abundance was assessed during two years. We predicted that exposure to episodic high concentrations of ozone during a series of growing cycles would result in plant assemblies with lower diversity (lower species richness and higher dominance), due to an increase in dominance of the stress tolerant species and the elimination of the ozone-sensitive species. As a consequence, arthropod-plant interactions would also be changed. Species richness of the recruited plant communities from different exposure histories was similar (≈ 15). However, the relative abundance of the dominant species varied according to history of exposure, with two annual species dominating ozone enriched plots (90 ppb: *Spergula arvensis*, and 120 ppb: *Calandrinia ciliata*). Being consistent both years, the proportion of carnivore species was significantly higher in plots with history of higher ozone concentration (≈3.4 and ≈7.7 fold higher in 90 ppb and 120 ppb plots, respectively). Our study provides evidence that, past history of pollution might be as relevant as management practices in structuring agroecosystems, since we show that an increase in tropospheric ozone may influence biotic communities even years after the exposure.

## Introduction

Human activities are changing not only Earth’s climate but also biotic communities at an unprecedented rate, modifying species range limits and causing extinctions from local to global scale [1]. The global atmospheric concentration of ozone (O_3_) in the troposphere has risen from less than 10 ppb (parts per billion) a century ago to 40 ppb today and is projected to continue to increase at an annual rate of 1–2% [2,3]. Great efforts are being done in different countries to decrease emissions of ozone precursors [4,5]. However it is difficult to estimate the needed emission reductions because of unknown long-term consequences on the exposed biota [6].

Ozone toxicity has long been believed to be mostly due to the formation of reactive oxygen species (ROS) such as superoxide, hydrogen peroxide, and hydroxyl radical resulting from O_3_ degradation in the cell apoplast [7]. Ozone effects at the plant level are often characterized as either acute or chronic responses. The former occur within hours after exposure to relatively high O_3_ concentrations (usually >150 nmol mol^-1^) [8]. As a result, cell death with typical foliar lesions from chlorosis and necrosis occur. In contrast, chronic responses include lesions that develop over days to weeks under lower O_3_ concentrations, and accelerated senescence mainly due to a decrease in photosynthetic efficiency [9]. Severe damage and productivity losses in crops and forests exposed to the pollutant have been observed [10,11].

Many species of plants acclimatize to elevated ozone relatively quickly, while others do not [12,13]. Populations may be directionally selected and become resistant to ozone [14,15]. Hence, there is a wide range of relative sensitivity to ozone among plant species, sometimes resulting in the elimination of ozone-sensitive species in polluted areas [16–18]. Free-radical-scavenging systems are thought to mediate the O_3_ resistance of plants [19]. Accordingly, some species gain relative advantage if their growth is not impaired under elevated ozone [20]. For example varying ozone exposures caused shifts in the competitive interactions between plants of an early successional plant community, thereby altering community structure [21].

Variation in vegetation texture can shape communities of herbivorous arthropods through effects on abundance, diversity, and distribution of their host plants [22]. In this context, an increase in tropospheric O_3_ may potentially affect plant-insect relationships indirectly through changes in the physical environment (e.g. plant architecture) or through altered plant biochemistry [23]. Ozone influences the biosynthesis of hormones and plant antioxidants able to improve not only the plant’s tolerance to environmental pollutants, but also the plant’s resistance to pathogens and herbivores [24,25]. Elevated O_3_ also interferes with plant-herbivore interactions through top-down effects on natural enemies of herbivorous arthropods, via shifts in the diversity, abundance and quality of prey or changes in host-finding mechanisms [26,27]. Natural enemies may be particularly sensitive to O_3_ through changes in searching behavior [28,29] or the behavior of their prey [30]. Ecological theory predicts that top down carnivore control of herbivores becomes more important for autotrophic persistence when environmental constraints caused by abiotic stressors are high [31]. For example, it has been documented that plants may have sophisticated adaptive mechanisms that allow them to attract carnivores, reducing the impact of herbivores on leaf area under stressful conditions, because compensatory growth to recover from damage is less likely in these scenarios [32,33]. Nevertheless the vast majority of literature on O_3_ effects addresses individual species responses, and very few have measured whole community responses. Moreover, higher trophic levels have been mostly ignored.

There is an increasing interest in understanding the impact of the history of land use on the present-day composition of plant communities in semi-natural grasslands [34–37]. However, the impact of global changes on the biology of species, rarely concentrate on more than one life cycle stage [38]. Seed-banks may store genetic information generated through acclimation and selection caused by previous environments [39–42]. Ageing, dormancy characteristics, and germination response of many weed species depend on the environment to which the mother plants were exposed [43,44]. In a previous study we showed that the selection pressure exerted on a weed species by long term exposure to tropospheric ozone, resulted in three populations with different seed behavior [45]. A differential synthesis of antioxidants was the proposed mechanism behind the prolonged seed viability in the soil. A weed community with a past history of pollution might establish after emissions of ozone precursors decrease, or if soil seed bank is transported together with crop seeds to a new low-pollution environment. Investigating how plant species diversity and associated arthropod community composition differ among stands that have different pollution histories can increase our understanding of the consequences of past pollution on agricultural communities. In this study we evaluated the legacy of four years of episodic ozone exposure on an herbaceous community of arable land. A plant ecosystem dominated by weeds, was exposed to three levels of ozone concentration for four years and seeds that naturally set from this experiment were eventually used to re-establish them under natural field environment in two years. In this way the effect of history of exposure to ozone episodes and storage time in the seed bank were tested by evaluating the structural and functional characteristics of the biological community that was established in a novel environment long after ozone exposure ended. We hypothesized that history of ozone pollution has an effect on the plant community. Exposure to episodic high concentrations of ozone during a series of growing cycles would result in plant assemblies with lower diversity (lower species richness and higher dominance), due to an increase in dominance of the stress tolerant species and the elimination of the ozone-sensitive species. We also hypothesized that the structure of the colonizing arthropod communities and the trophic interactions would be determined by the structural changes in plant community. We expected that the dominance of stress tolerant plant species would reduce herbivore diversity and alter top down interactions in the arthropod network.

## Materials and methods

## Long term ozone exposure experiment

Weed populations used in this work were selected in a long-term ozone exposure experiment carried out in Corvallis, Oregon, at the US Environmental Protection.

Agency Laboratory, Western Ecology Division (for details see Pfleeger et al [46]). This plant community was chosen because the plants are common to many areas of the world (mainly Eurasian annuals), they germinate readily, are easy to culture and are mainly annuals. Briefly soil containing a seed bank from an agricultural area was used. Nine open top chambers were used to expose the resulting plant community to one of three treatments (0, 90 and 120 ppb episodic ozone) for four consecutive years. Three chambers were used for the ‘control’ (0) treatment (charcoal-filtered air with low O_3_) and six for the O_3_ treatments (charcoal-filtered air with added O_3_). In the O_3_ treatments (90 and 120 ppb), ozone was generated from oxygen and added to the chambers. The ozone profile was developed based on the regional air quality data from the Midwest (USA) and consisted of an episodic pattern of varying daily peak concentration. Each chamber received the same episodic ozone exposure profile each year. Hourly requested peaks ranged from 1 to 155 ppb for the 90 episodic ozone treatments and 1 to 219 ppb for the 120 episodic ozone treatments. The high peaks lasted for 1 h [46]. At the end of the fourth season, 5 cm top soil containing seed bank of the community resulting from 4 years’ exposure to episodic ozone was removed from each chamber. Soil was air-dried at ambient temperature, and stored separately and refrigerated at 5°C in sealed plastic boxes until use.

### Field experiments

Samples of the seed bank were transferred into field plots and allowed a community to establish. Two separate experiments using the same original soil seed bank were conducted in two consecutive years. In mid-July each year, nine 1 m^2^ plots separated by 1 m all sides were established at the University of Buenos Aires, School of Agronomy experimental field (34°35’5” lat. S, 58°29’05” long. W). Each plot was dug approximately 25 cm and filled with previously sterilized soil. The soil seed banks belonging to each of the ozone chambers were randomly assigned to one of the nine plots each year, and a 5 cm layer of soil from each chamber was added to the top of the soil. A plant community was spontaneously established in each plot from the germination of seeds in the soil bank. The plots were isolated with 2 m tall transparent plastic walls during the reproductive period to prevent seed or pollen flow among plots, but allowing insect colonization.

### Measurements

Surveys were carried in the field plots to identify weed and arthropod communities. Each year in August four replicate 15 x 15 cm quadrats were placed in each plot. During the first month, number of seedlings per species was weekly recorded in each quadrat, after which species presence was monthly evaluated until December.

In order to determine the species diversity and abundance of the arthropod fauna associated with each plot, insects on the vegetation were sampled twice each year with a D-vac insect suction machine moved vertically for 1 min from the foliage to the ground surface over each plot except for a 10 cm border. Abundance of adult and immature stages of the collected arthropods were later determined in the lab by direct observation. Insect determination was done at order level in all cases and at family or species level when possible. To assess changes in the functional structure of the arthropod assemblages in response to past ozone exposure, arthropods were assigned to functional groups on the basis of larval feeding strategy as being predominantly carnivorous, herbivorous, or saprophagous (detritivorous). Carnivores and herbivores were further separated into more specialized groups.

### Data analysis

All analyses on community structure were based on the summed abundances of each plant and arthropod species for a particular year, as suggested in Colwell et al. [47]. For each year, samples were pooled across all sample dates. Each sampling date, some arthropods could not be identified, but were accounted for in the total number of individuals. Proportion of individuals corresponding to each plant species or insect guild was normalized using a log transformation. Treatment effects within each year were analyzed using two-way ANOVA with LSD *post hoc* tests. Species richness (the total number of species, *S*) and constancy (proportion of plots in which a given species occurred throughout all surveys) was calculated for every weed and insect in the community. Richness and specific abundance values were used to derive diversity index (Shannon-Weaver index, H´) as follows: *H*´ = -∑ *p*_i_ log_2_ *p*_i_, where *p*_i_ = n_i_/*N*: proportion of individuals in the *i*th species, *n*_i_ = number of individuals in each species and *N* = number of individuals. Species evenness was based upon the species diversity index calculated by *H*´/log_e_ (*S*) [48]. Treatments effects within each year were tested with one way ANOVA.

Repeated measures-ANOVA was used to evaluate treatment-related differences in the time-repeated measurement of seedling production during the evaluation period. Statistically significant differences between treatments for each species were localized with LSD *post hoc* tests. The relationship between arthropod diversity (H´) and plant species richness was tested with Spearman´s non-parametric correlation test. Results of P<0.05 were considered significant. Statistical analyses were conducted with InfoStat Professional Version 1.1.

## Results

### Plant community structure

Plant species richness in the plots set up in a common low-ozone environment in Argentina was highly reduced to over half the number of species historically recorded in the chambers under the different exposure regimes (Table 1). This reduction in species number was mainly explained by loss of legumes during seed bank storage in the soil.

**Table 1:** Community structure of the experimental plots during ozone exposure at 0, 90 and 120 ppb and after transplanting under common ecological conditions.

	During exposure ^a^				After exposure			
	0	90	120	*P*	0	90	120	*P*
Family richness	14	16	16	ns	11	9	11	ns
Legumes	32	31	33	ns	14	15	14	ns
Grasses	4	4	3	ns	3	4	3	ns
Other herbs	5	8	8	ns	2	3	3	ns

Values represent Mean number of families (richness) or species in each category during exposure (n = 12) and after exposure (n = 6). ns: not significantly different (*P* > 0.05) within each row

^a^ Pfleeger et al [46].

Species extinction during storage in the seed bank was not related to their constancy values in the chambers or the ozone treatment they characterized, *i*.*e*. all constancy and ozone treatment groups had some species that survived in the soil bank (S1 Table). Species richness in the field plots was similar both years after exposure, and it did not differ among plots corresponding to the different historical ozone exposure regimes. We also did not detect significant differences in diversity or evenness among ozone treatments in the field plots (Table 2).

**Table 2:** Means and statistics from analysis of variance of species richness (*S*), species diversity (Shannon-Weaver index, H´) and species evenness (*H*´/log_e_ (*S*)) for plant communities established from different historic exposure regimens.

Treatment	Species richness	Species diversity	Species evenness
**Year 1**			
0 ppb	13	0.51	0.20
90 ppb	16	0.64	0.23
120 ppb	12	0.64	0.24
*P*	0.19	0.77	0.62
**Year 2**			
0 ppb	15	0.37	0.14
90 ppb	12	0.62	0.24
120 ppb	17	0.73	0.26
*P*	0.33	0.66	0.62

Values represent Means (n = 3)

*P* > 0.05 not significant differences within each column for a particular year

Total seedling density and specific abundance in the field plots in both years was higher in samples coming from the chambers exposed to historic episodic ozone than in those from control chambers (Table 3).

**Table 3:** Mean seedling density in 1 m^2^ plots established from plant communities. selected under different episodic concentrations of tropospheric ozone (0, 90 and 120 ppb).

Species	Year 1			Year 2		
	0	90	120	0	90	120
*Spergula arvensis*	99.3a	158.3b	129b	104.6a	387b	216.6c
*Calandrinia ciliata*	12.6a	26.3b	40c	11.3a	114.3b	215.6c
*Medicago lupulina*	0	0.3	1	3.3	3.6	4
*Vicia tetrasperma*	0	0	0	1.3	1.3	0.3
*Rumex crispus*	0	0	0	1	0.6	0.3
*Polygonum persicaria*	1a	4b	1a	0	0	0
*Taraxacum officinale*	0.6	1.6	0	1	1	1
*Digitaria sanguinalis*	0.6	0.6	1	0.3	1	0.3
*Erodium cicutarium*	0	0.6	0.6	0.3	1	0.6
*Solanum sarachoides*	0	0.3	0.3	0	0	0
*Raphanus sativus L*.	0.6	0.6	0.3	0	0	0
*Echinochloa crusgalli*	0.3	0.3	0.3	0.3	0	0.6
*Chenopodium album*	0.3	0.3	0	0	0.3	0.6
*Trifolium repens*	0.3	1	0.3	1	0.6	0.6
*Polygonum argyrocoleon*	0.3	0.3	0.3	0	0	0
*Datura stramonium*	1	0.3	1	0	0	0
*Convolvulus arvensis*	0.3	0	0.3	0	0	0
*Tanacetum vulgare*	0	0.3	0.3	0	0	0
*Oxalis corniculata*	1	0.6	0.6	0	0	0
*Sonchus oleraceus*	0	0	0	0.3	0.3	0.3

Values represent mean number (n = 3)

Means followed by different letters within rows for a particular year are significantly different (*P* = 0.05)

**Fig 1:**
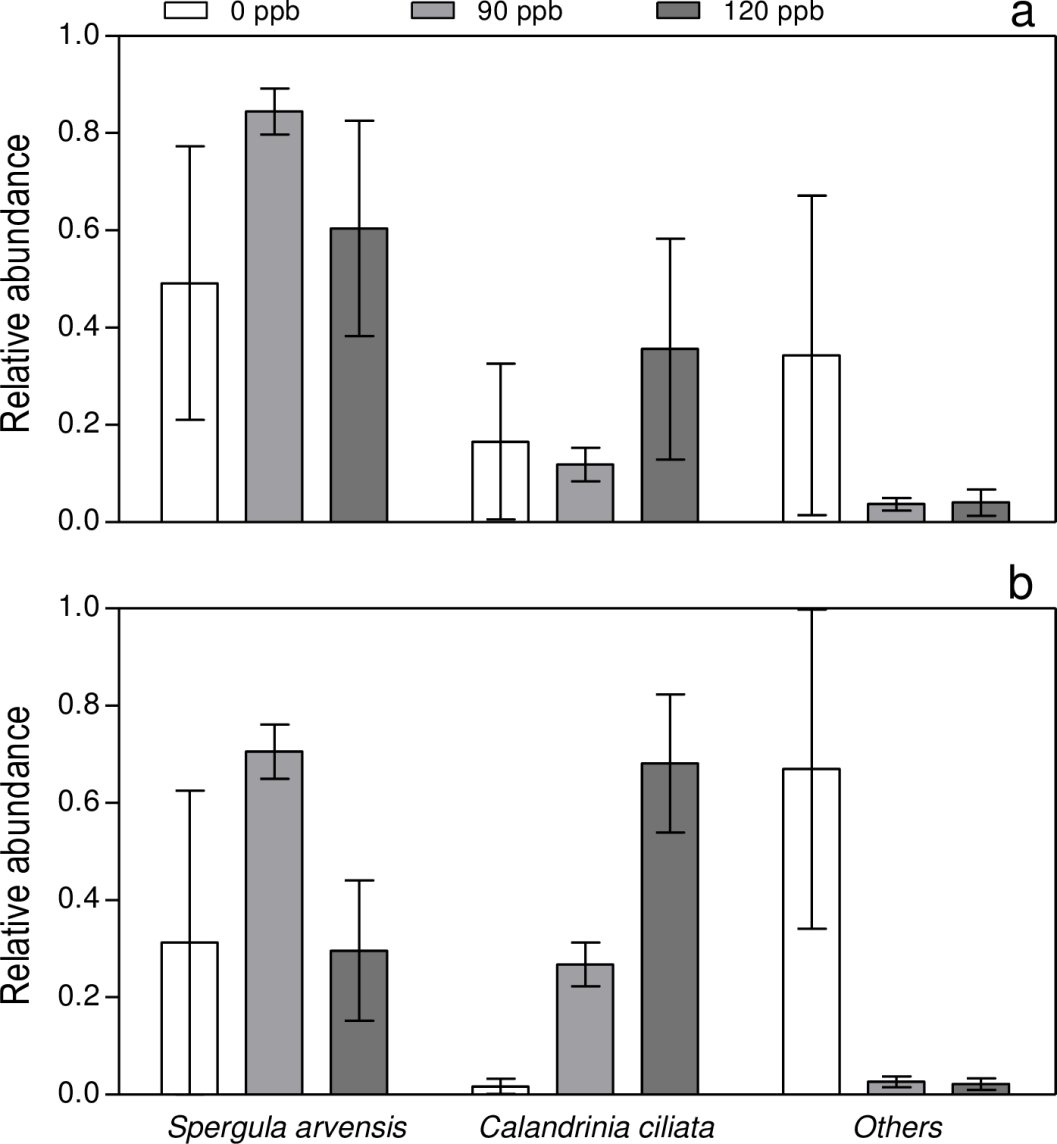
Relative abundance of *Spergula arvensis*, *Calandrinia ciliata* and other species in plant communities selected under different episodic concentrations of tropospheric ozone. Ozone concentrations in the open top chambers during long-term exposure were 0 ppb (white bars), 90 ppb (grey bars) and 120 ppb (dark bars). Data represents species abundances during the first (a) and second (b) year of experiment after original soil seed bank was transplanted to a common natural field environment. Relative abundance for each plant species was calculated as the summed abundances of each plant species for a particular year/total number of seedlings recorded in the plot (n = 3). Error bars represent standard error. Year a ANOVA *P*_species_ < 0.01, *P*_ozone_ 0.016, *P*_species x ozone_ 0.034; year b ANOVA *P*_species_ < 0.01, *P*_ozone_ 0.024, *P*_species x ozone_ 0.042

These differences were mainly caused by seedling density of the two dominant species *Spergula arvensis* and *Calandrinia ciliata* which increased their relative importance with the ozone exposure (Fig 1). On the other hand, high ozone levels depressed the other species (Fig 1).

### Arthropod community

Richness of spontaneous arthropod assemblies colonizing the plots was similar among treatments (Table 4). Eighty nine arthropod species were found in the field plots distributed among 58 families and 10 functional groups. Phloem-sucking (Hemiptera) was the most frequent group. Grouping according to constancy values (species presence over all surveys) allowed for the identification of distinct assemblies corresponding to the historic ozone exposure (S2 Table).

**Table 4:** Means and statistics from analysis of variance of species richness (*S*), species diversity (Shannon-Weaver index, H´) and species evenness (*H*´/log_e_ (*S*)) for arthropod communities established from different historic exposure regimens.

Treatment	Species richness	Species diversity	Species evenness
**Year 1**			
0 ppb	37	0.73	0.67
90 ppb	40	1.13	0.89
120 ppb	33	1.33	0.91
*P*	0.07	0.31	0.77
**Year 2**			
0 ppb	38	1.01	0.78
90 ppb	44	1.34	0.91
120 ppb	40	1.42	0.92
*P*	0.18	0.16	0.66

Values represent Means (n = 3)

*P* > 0.05 not significant differences within each column for a particular year

**Fig 2:**
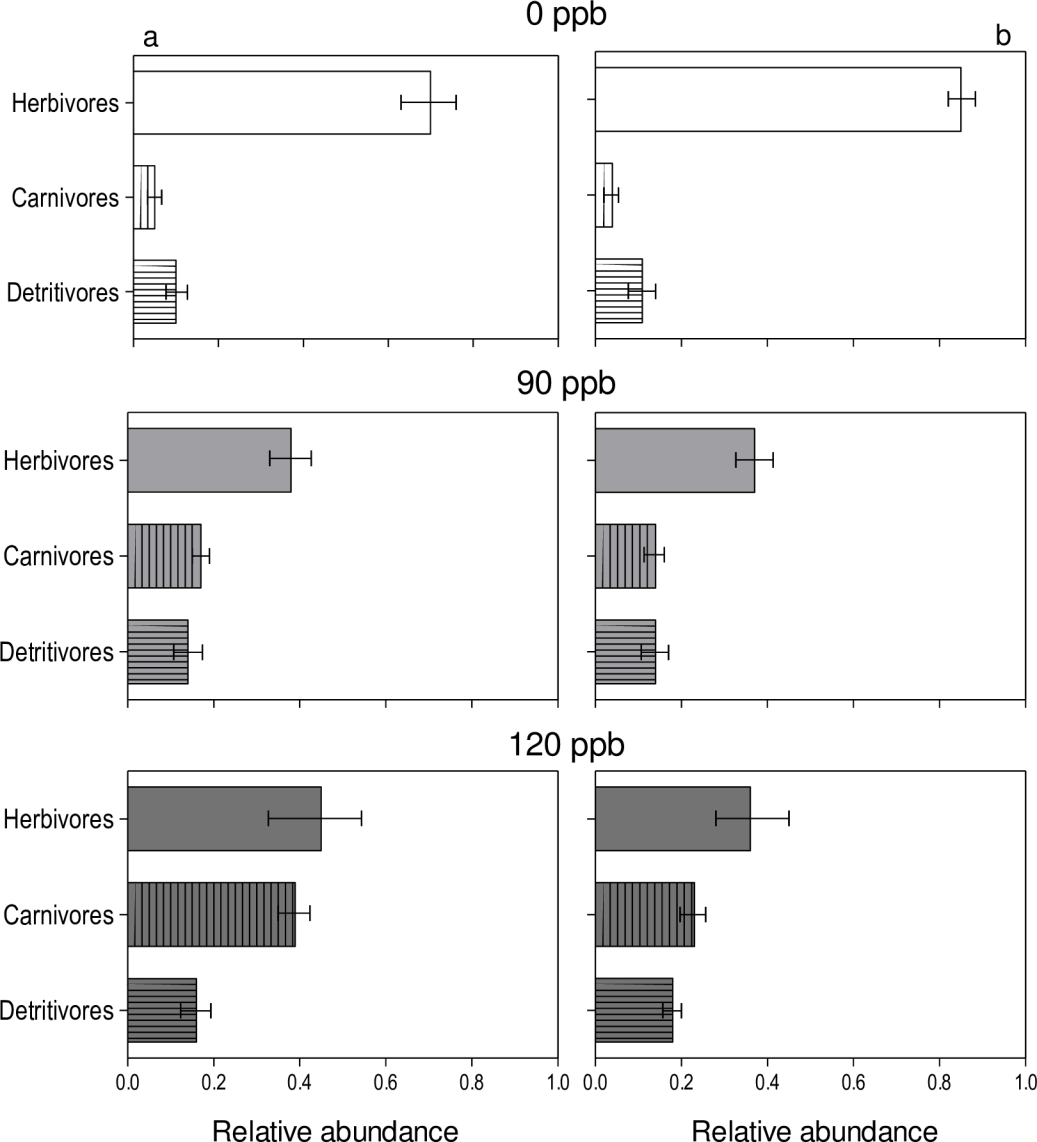
Relative abundance of arthropods corresponding to different functional groups colonizing field plots originated from plant communities selected under different episodic concentrations of tropospheric ozone. Ozone concentrations in the open top chambers during long-term exposure were 0 ppb (white bars), 90 ppb (grey bars) and 120 ppb (dark bars). Data represents arthropod abundances during the first (a) and second (b) year of experiment, after original soil seed bank was transplanted to a common natural field environment. Arthropods were assigned to different groups (guilds) according to larval feeding strategy. Relative abundance for each guild was calculated as the summed abundances of insects of that guild for a particular year/total number of insects recorded in the plot (n = 3). Error bars represent standard error. Year a ANOVA *P*_guild_ < 0.01, *P*_ozone_ 0.03, *P*_guild x ozone_ 0.021; year b ANOVA *P*_guild_ 0.02, *P*_ozone_ 0.014, *P*_guild x ozone_ 0.07

The proportion of each functional group was related to the ozone level to which the community had been exposed (Fig 2). Herbivore arthropods were especially sensitive to the different plant community structure generated by the ozone treatments. Both years the relationship carnivore/herbivore increased with historic ozone level (Fig 2).

**Fig 3:**
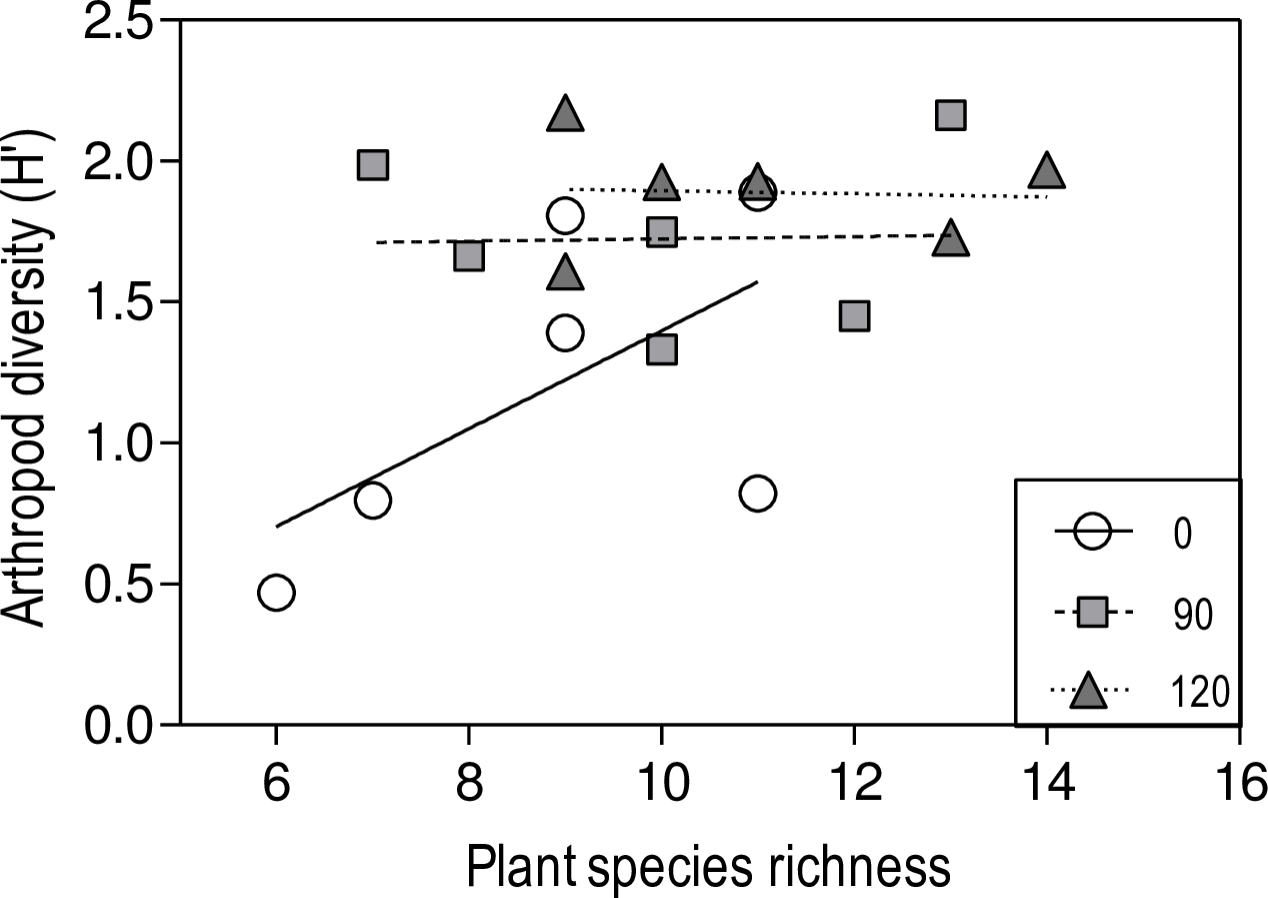
Relationship between the arthropod diversity (Shannon index H´) and the number of plant species. Plant species were recorded in the field plots where soil seed bank selected under different episodic ozone concentrations [0 (white circle), 90 (grey square) and 120 (black triangle) ppb] was sown. Arthropods naturally colonized the plots. Each data point corresponds to one plot coming from a particular ozone concentration in one of two years of experiment. Spearman´s correlation 0 ppb r_s_ = 0.71, n = 6, *P* < 0.01; 90 ppb r_s_ = 0.02, n = 6, *P* = 0.75; 120 ppb, r_s_ = 0.10, n = 6, *P* = 0.68

Linear regression models revealed a positive significant association between diversity of arthropods and plant richness in each control plot coming from non-exposed communities. However, this relationship was lost in plots with a history of ozone exposure (Fig 3).

## Discussion

We showed that long-term exposure to episodic ozone provided a legacy resulting in altered patterns of dominance in plant and arthropod communities. However historic ozone exposure did not modify species richness. Number of plant species decreased in the new ambient environment but it remained similar among the different plots corresponding to historic exposure. The different groups of dominant and subordinate species occurring in the ozone treatments, and the change in relative importance of the two dominant species, clearly shows a differential sensitivity for ozone among the species. Nevertheless, our results suggest that single species tests would have not allowed us to predict changes in community during exposure, or the ability of the species to resist storage in the soil seed bank. This is in accordance with recent results on long-term effects of ozone on forest ecosystems [49]. The researchers used a computer model to study how species-specific responses to ozone can change the competitive interactions among species. They found that ozone changes the relative abundances of tree species, but species richness, overall ecosystem productivity -the rate of biomass generation -and the ability of the ecosystem to store carbon do not change in the face of ozone pollution.

Pfleeger et al. [46] did not detect differences in reproduction or seedling emergence among plots exposed to different ozone levels. However it is apparent that historic ozone episodes increased the persistence of the seed-bank of several species, especially those of *C*. *ciliata* and *S*. *arvensis*. In a previous study, exposure to high ozone of *Lolium multiflorum* plants resulted in seeds with higher levels of glutathione, an antioxidant related to higher viability in seeds [50]. It is possibly that in our study, only those individuals that had the ability to produce enough antioxidant may have survived and reproduced, producing seeds with higher antioxidant concentration. In this way, the populations would adapt, not through a higher ozone tolerance, but through the selection of those individuals that produced seeds with the ability to remain alive in the soil for longer periods. Resilience of arable land communities is frequently attributed to the existence of persistent soil seed banks, and the importance of seed-banks to backup adaptive information of past environments has been repeatedly discussed [51–54]. In this experiment we were able to account for a long-term effect at the community level that resulted from repeated episodes of ozone exposure on the plant community stored in the seed bank.

Past history of ozone exposure was a strong determinant of the structure of the colonizing arthropod community. This pattern occurred despite plant richness not being affected by the ozone treatments. However, arthropod community structure was positively correlated with number of plant species as accounted by H´ index. This relationship is not surprising since plant and arthropod diversities are expected to vary in the same direction [55]. On the other hand, diversity of arthropod was higher and not related to plant richness in plots coming from historic ozone exposure. This suggests that the different arthropod assemblies observed in the ozone plots would be determined either by a different spatial arrangement of the species, or by different quality of the individuals of the plant dominant species. During the last decade several studies have proposed that the genetic variability of the lower trophic levels determines the higher level´s structure and thus the way in which communities are assembled [56–58]. Other studies have shown that the presence of a genetically diverse plant neighborhood induces changes in plant biomass [59] and impacts on the arthropod communities that colonize them [60]. Recent reviews have suggested that such changes might also be mediated by plant volatile signals [61–62]. Hence, variation in arthropod community may have been induced by particular ecological conditions generated by the characteristics of the individuals of the most abundant plant species, which were differentially selected depending on the ozone levels. This is supported by previous experiments in which we found that individuals of *C*. *ciliata* and *S*. *arvensis* that were obtained from seeds of the same seed-bank of this experiment, and grew in a common garden, had different structural and growth characteristics. Moreover, these plants expressed different damage levels when exposed to herbivores depending on the level of historic ozone exposure [63].

The food web structure of arthropod community showed marked differences associated to ozone historic level. In both years the carnivore to herbivore ratio had greater values with increasing levels of ozone historic episodes, suggesting that the plants that evolved under the high ozone stressful environment may over-express mechanisms of carnivore attraction. This in part explains the high arthropod diversity in these plots, even when plant diversity was low. The capacity to compensate for the cost of stress by improving their growth environment in ecosystem-scale has been demonstrated for forests and crops exposed to ozone pollution and other stress factors [64–67]. Under these conditions, an increase in VOCs production and reduction in herbivore attack has been observed. Future investigation will determine if this compensation mechanism could also operate as a result of evolutionary change. Together, the previous studies and our results suggest that increases in tropospheric ozone will have negligible impacts on arthropod species richness. However, persistent high levels of exposure might result in changes in food webs (proportion of feeding guilds) either via direct effect over the short term, or plant-mediated effects through evolutionary processes on the host plant populations.

## Conclusion

This study provides evidence that, past history of pollution might be as relevant as management practices in structuring biotic communities. We show that an increase in tropospheric ozone may influence biotic communities even years after the exposure, becoming a legacy that may determine to some extent, the pattern of response of the plant communities originated from persistent soil seed-banks, and the arthropod assemblies in a novel environment that occur when seeds germinate. This has implications for both interpreting data on how communities are structured, and to acknowledge that, even if the different actions that are being taken to minimize the impact of global change driven by human activities are successful, ecological consequences of increases in atmospheric pollution would be long-lasting.

## Supporting information

S1 Table Plant species ordered by constancy values during and after episodic ozone exposure. Constancy: the proportion of plots within a set of even-sized plots in which a certain species occurs.
(DOC)

S2 Table Arthropod families ordered by constancy in plots with communities established from soils with different exposure histories. Constancy: the proportion of plots within a set of even-sized plots in which a certain family occurs. Function: Herbivores: H-Chew (herbivore-chewing), H-Suc (herbivore-sucking), H-Nect (nectivore), Carnivores: B-Suc (blood-sucking), Fung (fungivore), Par (parasitoid), Zooph (zoophilo, animal secretion, sweat mainly), Car (other carnivores), Detritivores: Sapr (saprophagous), Scav (scavenger). Stage: development stage: L (larvae), A (adult)
(DOCX)
