# Supplementary material for: Legacy of historic ozone exposure on plant community and food web structure
Source: PLoS One. 2017 Aug 10;12(8):e0182796. doi: 10.1371/journal.pone.0182796 (PMC5552163; doi:10.1371/journal.pone.0182796)
Supplement: S1 Table — Constancy: the proportion of plots within a set of even-sized plots in which a certain species occurs. (DOC) [file pone.0182796.s001.doc]

**S1 Table:** Plant species ordered by constancy values during and after episodic ozone exposure.

|  | **Constancy during O_3_ exposure** | | | | **Constancy after O_3_ exposure** | | |  |
| --- | --- | --- | --- | --- | --- | --- | --- | --- |
| **Species** | **0 ppb** | **90 ppb** | **120 ppb** | **Total** | **0 ppb** | **90 ppb** | **120 ppb** |  |
| *Spergula arvensis* | 100 | 100 | 100 | 100 | 83 | 100 | 100 |  |
| *Calandrinia ciliata* | 100 | 100 | 100 | 100 | 67 | 100 | 100 |  |
| *Digitaria sanguinalis* | 100 | 100 | 100 | 100 | 83 | 50 | 83 |  |
| *Erodium cicutarium* | 100 | 100 | 100 | 100 | 33 | 67 | 67 |  |
| *Vicia tetrasperma* | 100 | 100 | 100 | 100 | 50 | 50 | 50 |  |
| *Sonchus sp.* | 100 | 100 | 100 | 100 | 50 | 50 | 33 |  |
| *Capsella bursa-pastoris* | 100 | 100 | 100 | 100 | 33 | 33 | 17 |  |
| *Solanum nigrum* | 100 | 100 | 100 | 100 | 33 |  | 33 |  |
| *Amaranthus powellii* | 100 | 100 | 100 | 100 |  |  | 33 |  |
| *Eragrostis orcuttiana* | 100 | 100 | 100 | 100 |  |  |  |  |
| *Panicum capillare* | 100 | 100 | 100 | 100 |  |  |  |  |
| *Veronica biloba* | 100 | 100 | 100 | 100 |  |  |  |  |
| *Poa annua* | 100 | 75 | 100 | 92 |  |  |  |  |
| *Senecio vulgaris* | 100 | 75 | 100 | 92 |  |  |  |  |
| *Stellaria media* | 75 | 100 | 100 | 92 |  |  |  |  |
| *Echinochloa crusgalli* | 100 | 75 | 75 | 83 | 33 | 33 | 50 |  |
| *Chenopodium ambrosoides* | 75 | 75 | 100 | 83 |  |  |  |  |
| *Oxalis corniculata* | 75 | 75 | 75 | 75 | 17 | 17 | 33 |  |
| *Solanum sarrachoides* | 75 | 75 | 75 | 75 |  | 33 | 33 |  |
| *Cerastium viscosum* | 75 | 75 | 75 | 75 |  |  |  |  |
| *Raphanus sativus* | 50 | 75 | 75 | 67 | 50 | 33 | 33 |  |
| *Euphorbia supina* | 50 | 75 | 75 | 67 |  |  |  |  |
| *Medicago lupulina* | 50 | 50 | 75 | 58 | 50 | 67 | 67 |  |
| *Polygonum persicaria* | 50 | 50 | 50 | 50 | 17 | 33 | 17 |  |
| *Epilobium watsonii* | 50 | 50 | 50 | 50 |  |  |  |  |
| *Poa bulbosa* | 50 | 50 | 50 | 50 |  |  |  |  |
| *Trifolium ciliatum* | 50 | 50 | 50 | 50 |  |  |  |  |
| *Trifolium microcephalum* | 50 | 50 | 25 | 42 |  |  |  |  |
| *Agrostis stolonifera* | 25 | 25 | 50 | 33 |  |  |  |  |
| *Rorippa curvisilique* | 25 | 25 | 50 | 33 |  |  |  |  |
| *Trifolium hybridum* | 25 | 25 | 50 | 33 |  |  |  |  |
| *Taraxacum officinale* | 25 | 25 | 25 | 25 | 83 | 50 | 50 |  |
| *Barbarea orthoceras* | 25 | 25 | 25 | 25 |  |  |  |  |
| *Hypochaeris radicata* | 25 | 25 | 25 | 25 |  |  |  |  |
| *Anthemis cotula* | 25 | 50 | 75 | 50 |  |  |  |  |
| *Polygonum argyrocoleon* | 25 | 50 | 100 | 58 | 17 | 33 | 50 |  |
| *Convolvulus arvensis* |  | 75 | 75 | 50 | 17 |  |  |  |
| *Rumex crispus* |  | 25 | 75 | 33 | 33 | 33 | 50 |  |
| *Datura stramonium* |  | 50 | 50 | 33 | 33 | 17 | 17 |  |
| *Chenopodium album* | 50 | 100 | 100 | 83 | 50 | 50 | 17 |  |
| *Arabidopsis thaliana* | 50 | 100 | 100 | 83 | 17 | 33 | 33 |  |
| *Lolium multiflorum* | 100 | 75 | 25 | 67 |  |  |  |  |
| *Portulaca oleracea* | 100 | 75 | 25 | 67 |  |  |  |  |
| *Phalaris arundinacea* | 75 |  |  | 25 |  |  |  |  |
| *Tanacetum vulgare* | 50 |  |  | 17 |  | 50 | 17 |  |
| *Lotus micranthus* | 50 |  |  | 17 |  |  |  |  |
| *Poa gracillima* | 25 |  |  | 8 |  |  |  |  |
| *Poa trivialis* | 25 |  |  | 8 |  |  |  |  |
| *Veronica heredaefolia* | 25 |  |  | 8 |  |  |  |  |
| *Lamium amplexicaule* | 50 | 50 |  | 33 |  |  |  |  |
| *Cyperus sp.* | 25 | 50 |  | 25 |  |  |  |  |
| *Melilotus albus* | 25 | 25 |  | 17 | 17 | 17 | 17 |  |
| *Panicum dichotomiflorum* | 25 | 25 |  | 17 |  |  |  |  |
| *Trifolium repens* | 25 | 25 |  | 17 | 83 | 50 | 33 |  |
| *Allium textile* | 25 |  | 25 | 17 |  |  |  |  |
| *Verbascum thapus* | 25 |  | 25 | 17 |  |  |  |  |
| *Phalaris caroliniana* | 25 |  | 25 | 17 |  |  |  |  |

Constancy: the proportion of plots within a set of even-sized plots in which a certain species occurs
