# Supplementary material for: Legacy of historic ozone exposure on plant community and food web structure
Source: PLoS One. 2017 Aug 10;12(8):e0182796. doi: 10.1371/journal.pone.0182796 (PMC5552163; doi:10.1371/journal.pone.0182796)
Supplement: S2 Table — Constancy: the proportion of plots within a set of even-sized plots in which a certain family occurs. Function: Herbivores: H-Chew (herbivore-chewing), H-Suc (herbivore-sucking), H-Nect (nectivore), Carnivores: B-Suc (blood-sucking), Fung (fungivore), Par (parasitoid), Zooph (zoophilo, animal secretion, sweat mainly), Car (other carnivores), Detritivores: Sapr (saprophagous), Scav (scavenger). Stage: development stage: L (larvae), A (adult) (DOCX) [file pone.0182796.s002.docx]

**S2 Table:** Arthropod families ordered by constancy in plots with communities established from soils with different exposure histories.

| **Order** | **Family**  **(subfamiliy)**  **SO: suborder** | **Stage** | **Function** | | **Constancy** | | | | | | |
| --- | --- | --- | --- | --- | --- | --- | --- | --- | --- | --- | --- |
|  |  |  |  |  | **0 ppb** | | **90 ppb** | | **120 ppb** | | **Total** |
| Collembola | Entomobryidae | L-A | Sapr | | 66 | | 66 | | 66 | | 66 |
| Hemiptera | Aphididae | L-A | H-Suc | | 50 | | 83 | | 50 | | 61 |
| Hemiptera | Lygueidae | L-A | H-Suc | | 41 | | 25 | | 66 | | 44 |
| Hemiptera | Myridae | L-A | H-Suc | | 41 | | 33 | | 41 | | 38 |
| Araneae | Several | L-A | Car | | 41 | | 33 | | 41 | | 38 |
| Hemiptera | Cicadellidae | L-A | H-Suc | | 58 | | 33 | | 17 | | 36 |
| Diptera | Sciaridae | A | Nect | | 25 | | 41 | | 25 | | 30 |
| Hymenoptera | (Myrmicinae) | A | Scav | | 25 | | 41 | | 25 | | 30 |
| Diptera | Agromyzidae | L-A | Nect | | 8 | | 41 | | 41 | | 30 |
| Hemiptera | Myridae | L-A | H-Suc/Car | | 17 | | 25 | | 33 | | 25 |
| Hymenoptera | (Formicinae) | A | Scav | | 25 | | 33 | | 17 | | 25 |
| Coleoptera | Coccinelidae | L-A | Car | | 41 | | 8 | | 17 | | 22 |
| Coleoptera | Pselaphidae | A | Car | | 8 | | 8 | | 25 | | 14 |
| Diptera | Chironomidae | A | Nect | | 25 | | 8 | | 8 | | 14 |
| Hemiptera | Delphacidae | A | H-Suc | | 8 | | 17 | | 8 | | 11 |
| Diptera | Sphaeroceridae | A | Nect | | 17 | | 8 | | 8 | | 11 |
| Diptera | Ephydridae | A | Nect | | 17 | | 8 | | 8 | | 11 |
| Diptera | Chloropidae | A | Zooph | | 8 | | 17 | | 8 | | 11 |
| Hymenoptera | Braconidae | A | Par | | 8 | | 17 | | 8 | | 11 |
| Acari | Mesostigmata | L-A | Sapr | | 8 | | 8 | | 17 | | 11 |
| Hymenoptera | (Dolichoderinae) | A | Scav/Sapr | | 8 | | 8 | | 8 | | 8 |
| Diptera | Ceratopogonidae | A | Nect | |  | | 25 | | 17 | | 14 |
| Hymenoptera | Aphidiinae | A | Par | |  | | 17 | | 25 | | 14 |
| Hemiptera | Fulgoridae | A | H-Suc | |  | | 8 | | 8 | | 5 |
| Coleoptera | Curculionidae | A | H-Chew | |  | | 8 | | 8 | | 5 |
| Coleoptera | Chrysomelidae | L | H-Chew | |  | | 8 | | 8 | | 5 |
| Hymenoptera | Braconidae | A | | Par | |  | | 8 | | 8 | 5 |
| Hymenoptera | Pteromalidae | A | | Par | |  | | 8 | | 8 | 5 |
| Orthoptera | Acrididae | L | | H-Chew | | 17 | | 8 | |  | 8 |
| Diptera | Cecydomidae | A | | Nect | | 17 | | 8 | |  | 8 |
| Diptera | Dolichoporidae | A | | Car | | 17 | | 8 | |  | 8 |
| Acari | Oribatidae | L-A | | Sapr | | 8 | | 17 | |  | 8 |
| Hemiptera | Aleyrodidae | A | | H-Suc | | 8 | | 8 | |  | 5 |
| Thysanoptera | Thrypidae | A | | H-Suc | | 17 | |  | | 17 | 11 |
| Hymenoptera | Figitidae | A | | Par | | 8 | |  | | 8 | 5 |
| Orthoptera | Tettigonidae | L-A | | H-Chew | | 17 | |  | |  | 6 |
| Hymenoptera | Scelonidae | A | | Par | | 25 | |  | |  | 8 |
| Psocoptera | Psocidae | A | | H-Suc | | 8 | |  | |  | 3 |
| Hemiptera | Berytidae | A | | H-Suc | | 8 | |  | |  | 3 |
| Coleoptera | Carabidae | A | | Car | | 8 | |  | |  | 3 |
| Coleoptera | Riphiphoridae | A | | Sapr | | 8 | |  | |  | 3 |
| Diptera | Phoridae | A | | Nect | | 8 | |  | |  | 3 |
| Diptera | Anthomyiidae | A | | Nect | | 8 | |  | |  | 3 |
| Diptera | Platystomatidae | A | | Nect | | 8 | |  | |  | 3 |
| Hymenoptera | Proctotrupidae | A | | Par | | 8 | |  | |  | 3 |
| Acari | Oribatida | A | | Sapr | | 8 | |  | |  | 3 |
| Lepidoptera | Yponomeutidae | L-A | | H-Chew | |  | |  | | 17 | 6 |
| Mantodea | Mantidae | L | | Car | |  | |  | | 8 | 3 |
| Odonata | SO Anisoptera | L | | Car | |  | |  | | 8 | 3 |
| Psocoptera | Stenopsocidae | A | | Fung | |  | |  | | 8 | 3 |
| Coleoptera | Coccinelidae | A | | Car | |  | |  | | 8 | 3 |
| Diptera | Mycetophilidae | A | | Nect | |  | |  | | 8 | 3 |
| Diptera | Syrphidae | A | | Nect | |  | |  | | 8 | 3 |
| Hymenoptera | Braconidae | A | | Par | |  | |  | | 8 | 3 |
| Hymenoptera | Myrmaridae | A | | Par | |  | |  | | 8 | 3 |
| Hymenoptera | Eupelmidae | A | | Par | |  | |  | | 8 | 3 |
| Hemiptera | Dipsocoridae | A | | Sapr | |  | | 8 | |  | 3 |
| Hemiptera | Reduvidae | A | | Car | |  | | 8 | |  | 3 |
| Hemiptera | Pentatomidae | A | | H-Suc | |  | | 8 | |  | 3 |
| Coleoptera | Carabidae | A | | Car | |  | | 8 | |  | 3 |
| Coleoptera | Lycidae | A | | Car | |  | | 8 | |  | 3 |
| Diptera | Dixidae | A | | B-Suc | |  | | 8 | |  | 3 |
| Diptera | Agromyzidae | A | | Nect | |  | | 8 | |  | 3 |
| Hymenoptera | Eulophidae | A | | Par | |  | | 8 | |  | 3 |
| Hymenoptera | Cynipidae | A | | Par/H | |  | | 8 | |  | 3 |

Constancy: the proportion of plots within a set of even-sized plots in which a certain family occurs

Function: Herbivores: H-Chew (herbivore-chewing), H-Suc (herbivore-sucking), H-Nect (nectivore), Carnivores: B-Suc (blood-sucking), Fung (fungivore), Par (parasitoid), Zooph (zoophilo, animal secretion, sweat mainly), Car (other carnivorous), Detritivores: Sapr (saprophagous), Scav (scavenger)

Stage: development stage: L (larvae), A (adult)
